# A mixed phosphine sulfide/selenide structure as an instructional example for how to evaluate the quality of a model

**DOI:** 10.1107/S2056989023002700

**Published:** 2023-03-28

**Authors:** Sean Parkin, Jeremy Cunningham, Brian Rawls, John E. Bender, Richard J. Staples, Shannon M. Biros

**Affiliations:** a Department of Chemistry, University of Kentucky, Lexington, KY, 40506, USA; b Department of Chemistry, Grand Valley State University, Allendale, MI 49401, USA; c Center for Crystallographic Research, Department of Chemistry, Michigan State University, East Lansing, MI 48824, USA; Universidad de Los Andes Mérida, Venezuela

**Keywords:** checkCIF alerts, *F* ^2^_obs_ *vs F* ^2^_calc_, OMIT command, outliers, displacement ellipsoids, solid solution, disordered electron density, standard inter­atomic distances, crystal structure

## Abstract

This paper compares variations on a structure model derived from an X-ray diffraction data set from a solid solution of chalcogenide derivatives of *cis*-1,2-bis­(di­phenyl­phosphan­yl)ethyl­ene, namely, 1,2-(ethene-1,2-di­yl)bis­(di­phenyl­phoshpine sulfide/selenide), C_26_H_22_P_2_S_1.13_Se_0.87_. A sequence of processes are presented to ascertain the composition of the crystal, along with strategies for which aspects of the model to inspect to ensure a chemically and crystallographically realistic structure. Criteria include mis-matches between *F*_obs_^2^ and *F*_calc_^2^, plots of |*F*_obs_| *vs* |*F*_calc_|, residual electron density, *checkCIF* alerts, pitfalls of the OMIT command used to suppress ill-fitting data, comparative size of displacement ellipsoids, and critical inspection of inter­atomic distances. Since the structure is quite small, solves easily, and presents a number of readily expressible refinement concepts, we feel that it would make a straightforward and concise instructional piece for students learning how to determine if their model provides the best fit for the data and show students how to critically assess their structures.

## Abstract 2

A sulfide/selenide solid-solution crystal structure is presented with an emphasis on model building and refinement. Some strategies and statistics for how to assess the accuracy of alternate models are described, including pitfalls, in the context of an instructional example that could be used as an activity in a classroom setting.

## Chemical context

**Figure 1:**
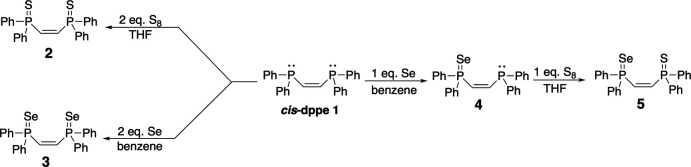
A generalized reaction scheme to prepare the compounds studied in this work.

Our research group has synthesized a number of phosphine chalogenide derivatives and explored their chemistry in regard to their coordination with both *d*-block and *f*-block metals (Luster *et al.*, 2022 ▸; Mugemana *et al.*, 2018 ▸; Morse *et al.*, 2016 ▸; Neils *et al.*, 2022 ▸). A few years ago, we worked with the rigid diphosphine *cis*-1,2-bis­(di­phenyl­phosphine)ethyl­ene **1** (*cis*-dppe), and developed conditions for the synthesis of the di-sulfide **2** (Rawls *et al.*, 2023 ▸) and the di-selenide **3** (Jones *et al.*, 2015 ▸) (Fig. 1 ▸). We obtained X-ray diffraction data for both **2** and **3**, and the structures were isomorphic, having the symmetry of the ortho­rhom­bic space group *P*2_1_2_1_2_1_. Our synthetic efforts then turned to preparation of mono-selenide **4** as a way to gain access to the mixed sulfide-selenide system, **5**. 
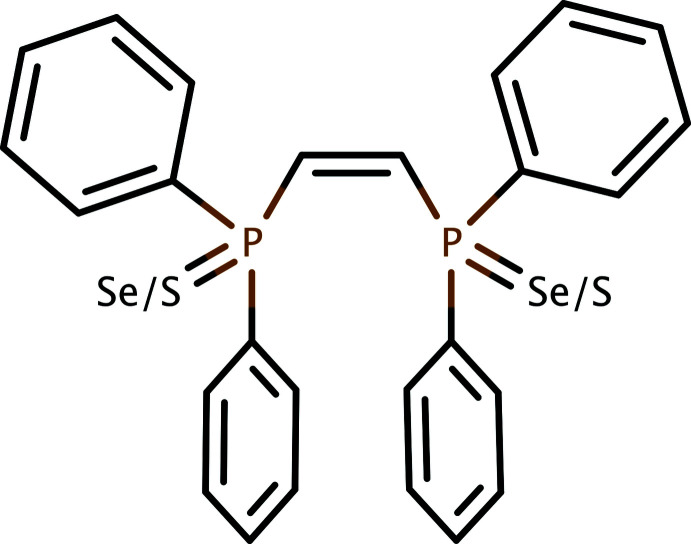


## Structure solution and model building trials

We obtained diffraction data for crystals grown directly from the reaction mixture. The structure solved easily in *P*2_1_2_1_2_1_ using *SHELXT* (Sheldrick, 2015*a* ▸) with included scattering factors for C, H, P, Se and a unit cell that was clearly related to those of compounds **2** and **3**. In the initial solution, however, *SHELXT* had assigned scattering factors for phospho­rus to both chalcogen sites, a chemical impossibility. In terms of similarity of scattering factors, sulfur would be the obvious next choice for the chalcogen, but the pnictogen-to-chalcogen distances (*vide infra*) were much longer than in the di-sulfide structure **2** (Rawls *et al.*, 2023 ▸). Given the available electron density at the chalcogen site, a disordered mono-selenide model seemed plausible. Of paramount importance here is that any trial model must be chemically plausible, thus knowledge of chemistry and information from other spectroscopic techniques (if available) should be used to rule out alternatives.

### Trial 1: a mono-selenide model

**Figure 2:**
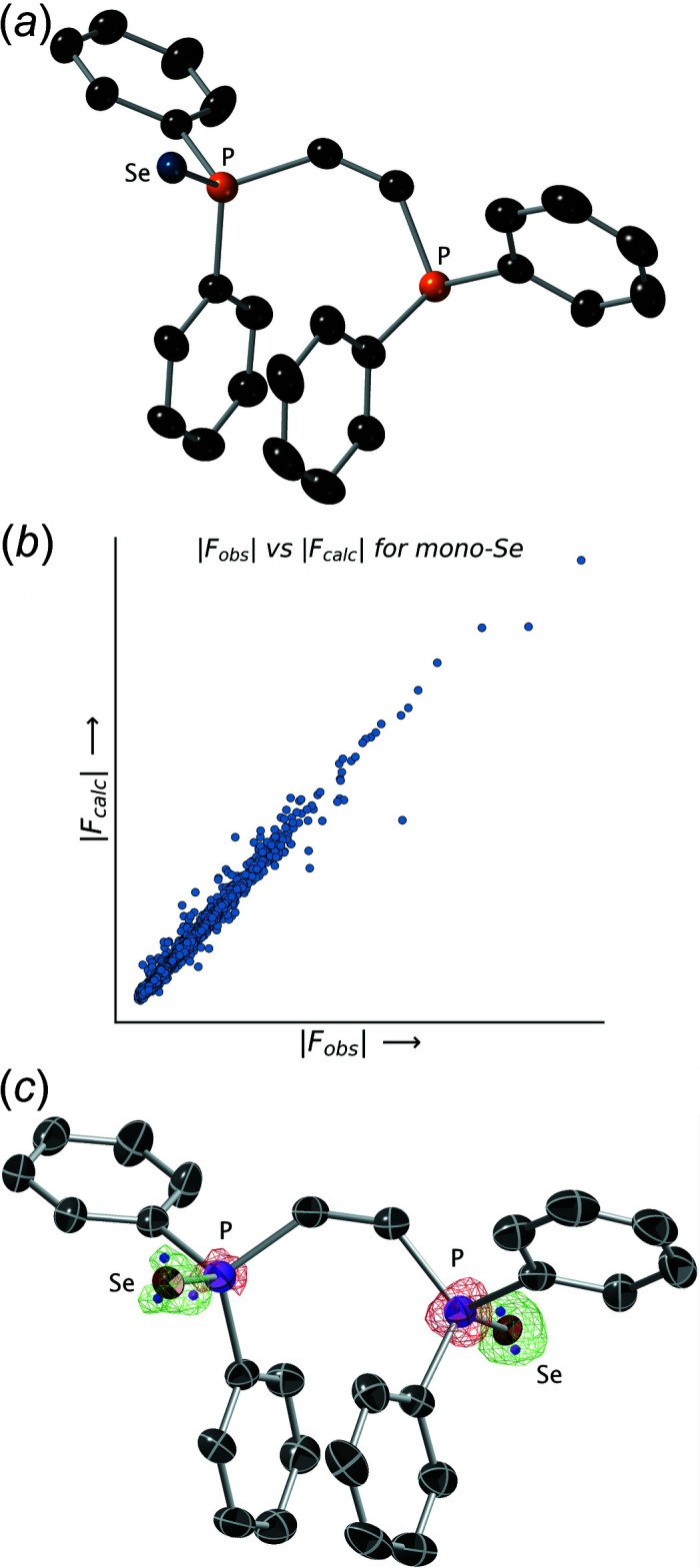
(*a*) An ellipsoid (50% probability) plot for the major component of the mono-selenide model. (*b*) A plot of |*F*_obs_| *vs* |*F*_calc_| values for the mono-selenide model. Note the extent of scatter in the plot. (*c*) A difference-electron density map reveals positive (green) electron density at the chalcogen site and negative (red) contours at the phospho­rus sites. Small peaks within the positive density are represented by coloured dots. The single Se atom in trial 1 is split with occupancies of 0.526 (2) and 0.474 (2) over the two chalcogen sites.

Manual editing of the chalcogen sites to accommodate a single Se atom split over the two sites gave a model that refined smoothly using *SHELXL* (Sheldrick, 2015*b* ▸), giving a disordered Se occupancy ratio of 0.526 (2):0.474 (2) (Fig. 2 ▸ *a*). The P=Se distances were 2.063 (2) and 2.035 (2) Å, and the model converged to an *R*_1_ value of 0.0494.

This model passed all the typical *checkCIF* tests, apart from returning a *B*-level alert indicating the presence of a few reflections with a poor fit between *F*_obs_^2^ and *F*_calc_^2^. The *SHELXL* list of ‘most disagreeable reflections’ (*i.e*, mis-matches between observed and calculated data) showed a striking difference for four reflections (Table 1 ▸, Fig. 2 ▸ *b*). At this point, it might be tempting to simply remove the top few poorly fitting reflections (*i.e.*, those having error/s.u. > 10) using the OMIT command in *SHELXL* and be satisfied with the structure. However, *all* of the worst outliers (see Table 1 ▸) have *F*_obs_^2^ >> *F*_calc_^2^ and are at resolutions far removed from the beamstop shadow. Thus, the commonly blamed culprit of ‘obscured by the beamstop’ would not provide any justification for omission. Unfortunately, uncritical omission of the worst-offending reflections in order to suppress unfavourable *checkCIF* alerts has become all too common. When *F*_obs_^2^ >> *F*_calc_^2^, however, such ill-fitting intensities are precisely the datapoints that are most sensitive to any model deficiencies. Modern data-reduction software includes facilities to identify the particular frame for any such outliers for manual inspection. In the present case, the offending reflections appeared to have been measured properly; no justification for omission was apparent. A closer look at the model showed that the displacement ellipsoids for the Se atoms were a little on the small side relative to neighbouring atoms, and that the residual difference-Fourier map showed pronounced electron density with several small embedded difference-map peaks (each less than 0.9 e Å^−3^) clustered near the chalcogen sites (Fig. 2 ▸ *c*). These ‘features’ suggest that this model did not fully account for all the electron density present in the data.

### Trial 2: a di-selenide model

**Figure 3:**
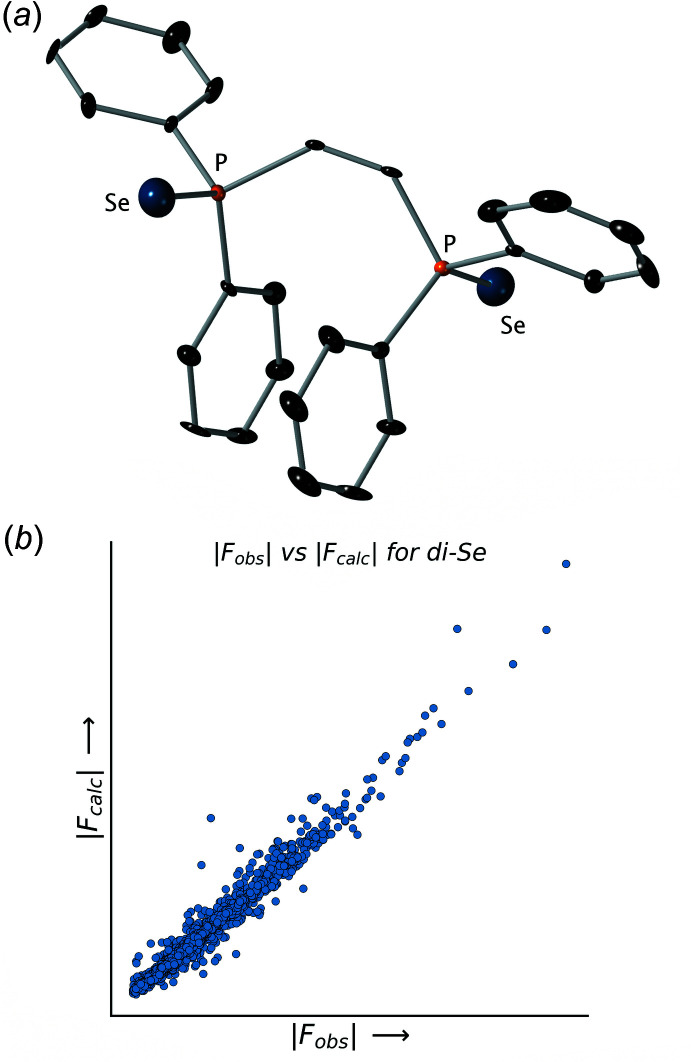
(*a*) An ellipsoid (50% probability) plot for the di-selenide model. Note the unrealistically large ellipsoids at the chalcogen sites and the small highly eccentric ellipsoids for the carbon atoms. (*b*) A plot of |*F*_obs_| *vs* |*F*_calc_| values for the di-selenide model. Note the extent of scatter in the plot.

To better account for the residual electron density, we built a model that corresponded to the di-selenide **3**, in which each Se atom had a fixed occupancy of 1.0 (Table 2 ▸ and Fig. 3 ▸). The resulting P=Se distances were (unsurprisingly) similar to those for the previous model at 2.061 (2) and 2.030 (2) Å. However, the *R*_1_ value for this model jumped to 0.0632 and the mis-match between the values of the displacement parameter tensors for the Se atoms and those for the rest of the atoms in the structure became wholly unrealistic (Fig. 3 ▸ *a*). Moreover, the discrepancy between the top four ‘disagreeable’ reflections became even larger, and the |*F*_obs_| *vs* |*F*_calc_| plot was a little more scattered (Fig. 3 ▸ *b*). By any measure, the di-selenide model is demonstrably worse than the mono-selenide model. Comparison of these two models, however, suggests that the electron density for the chalcogen atom sites present in the crystal that produced the diffraction data was insufficient to support two fully occupied selenium atoms, but it was too much for a mono-selenide model.

### Trial 3: a mono-selenide/di-selenide solid-solution model

**Figure 4:**
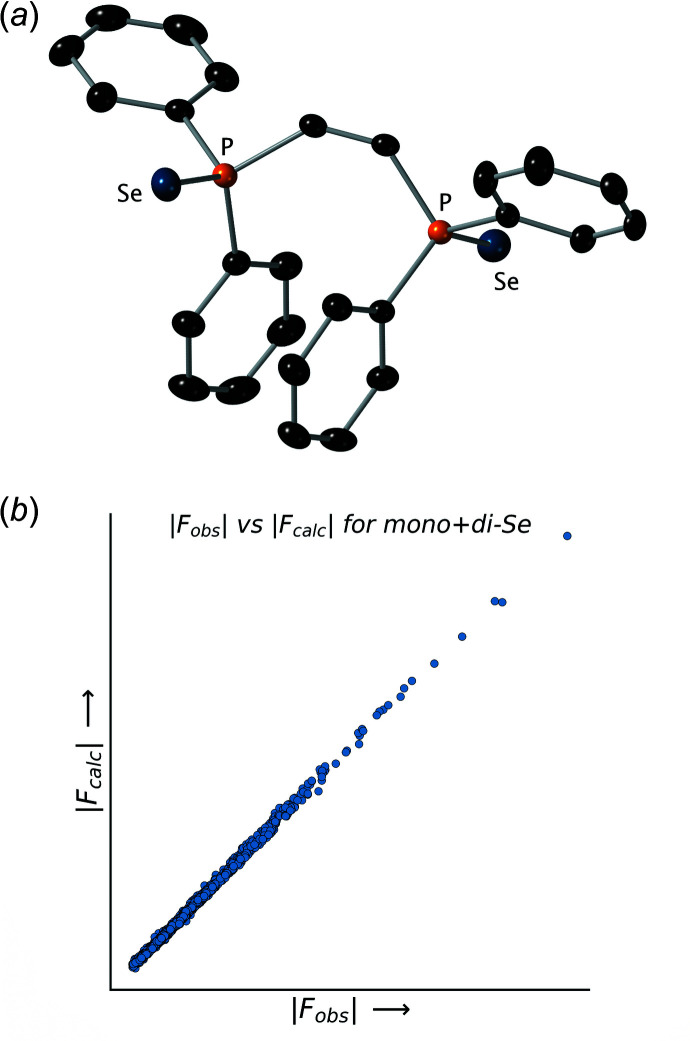
(*a*) An ellipsoid (50% probability) plot for the mixed mono-selenide/di-selenide model. Note that all ellipsoids appear quite normal. (*b*) A plot of |*F*_obs_| *vs* |*F*_calc_| values for the mixed mono-selenide/di-selenide model. Note the much reduced scatter in the plot relative to Figs. 2 ▸ and 3 ▸.

Since atomic scattering factors are (to a first approximation) proportional to atomic number, refinement of the occupancies at the chalcogen sites should give a good estimate of the amount of available density. Thus, to better fit the available electron density, the occupancies at each Se atom were refined freely, which gave occupancies of 0.712 (2) and 0.655 (2) for the two chalcogen sites (Fig. 4 ▸ *a*). The *R*_1_ value dropped quite precipitously to 0.0236 and the displacement ellipsoids for all atoms appeared to be acceptable. For this model, the *checkCIF* report revealed no *B*-level alerts, and the discrepancy between the top four observed *vs* calculated mis-matches was correspondingly *much* smaller than for any of the previous models (Table 3 ▸ and Fig. 4 ▸ *b*).

### Trial 4: a mixed selenide/sulfide solid-solution model

At this point, the statistics for the model were acceptable and *checkCIF* raised no red flags. Nonetheless, a critical comparison of the P=Se distances in the model shown in Fig. 4 ▸ *a* to values listed in *Inter­national Tables for Crystallography* vol. C (Table 9.5.1.1; Prince, 2006 ▸) and updated information in the CSD (Groom *et al.*, 2016 ▸) available *via MOGUL* (Bruno *et al.*, 2004 ▸) revealed additional *chemical* evidence that the outwardly acceptable model was subtly flawed. The average length for P=Se bonds is listed in these resources as 2.093 Å, while that of P=S bonds is 1.954 Å. The lengths of the P=Se bonds in the model shown in Fig. 4 ▸ *a* are in between these two values at 2.060 (8) and 2.0328 (8) Å. This observation is reminiscent of work by Gerard Parkin and co-workers in de-bunking the bond-stretch isomerism theory (Parkin, 1992 ▸), in which improbable ‘bond lengths’ were shown to result from undiagnosed chemical inhomogeneity rather than unrealistic bond-length differences.

**Figure 5:**
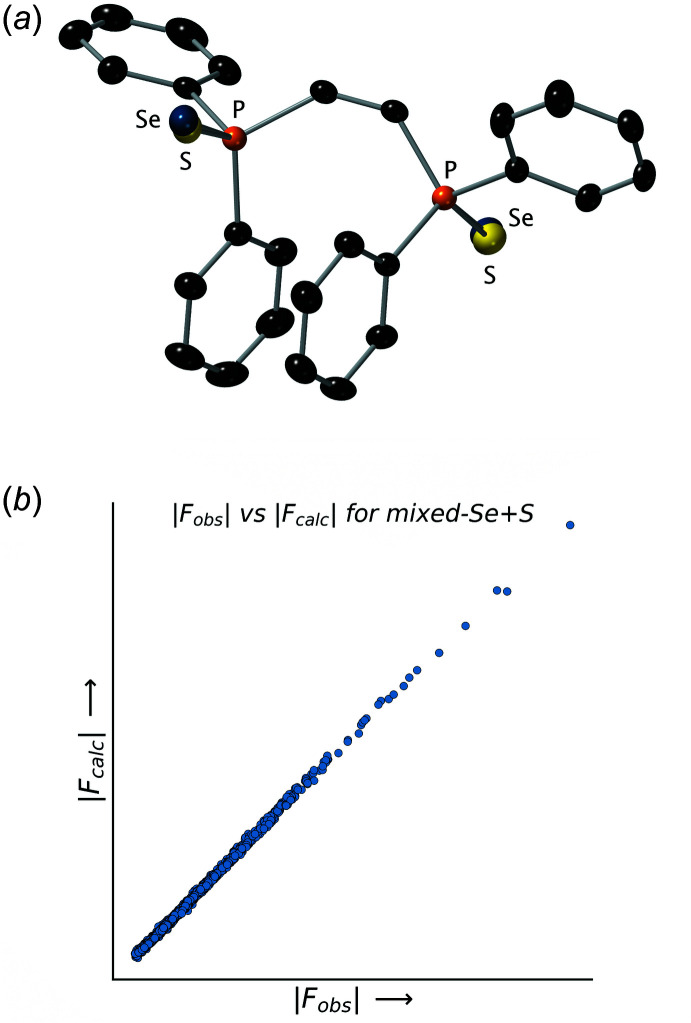
(*a*) An ellipsoid (50% probability) plot for the mixed selenide/sulfide model. Note that all ellipsoids appear quite normal. (*b*) A plot of |*F_obs_* | *vs* |*F_calc_* | values for the mixed selenide/sulfide model. Note the much reduced scatter in the plot relative to Figs. 2 ▸ and 3 ▸.

**Figure 6:**
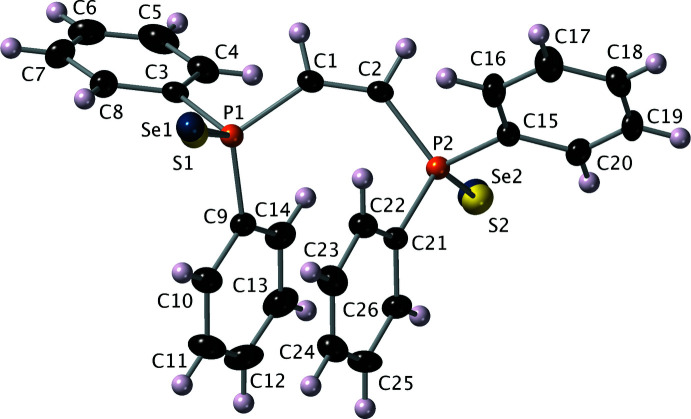
An ellipsoid (50% probability) plot for the final mixed selenide/sulfide model showing the atom-numbering scheme.

In a similar vein, one logical explanation for the discrepancy in the present case was that the sample could have been contaminated with some di-sulfide **2**. These compounds were all synthesized several years ago, and the mixed Se/S compound had been one of the synthetic goals (*vide supra*). Modification of the model to include selenium and sulfur at both sites, where the occupancies of these atoms were refined to sum to unity, resulted in the model shown in Fig. 5 ▸ *a* and 6. This model has an *R*_1_ value of 0.0209, notably lower than the previous selenide-only model, with the relative sulfur-to-selenium occupancies for each site being 0.513 (3):0.487 (3) and 0.614 (3):0.386 (3). The bond lengths for this final model are also in reasonable agreement with literature averages, the P=Se distances being 2.082 (8) and 2.088 (11) Å, while the P=S distances are 2.021 (19) and 1.953 (16) Å, directly from refinement, *i.e.*, *without* distance restraints. Since the literature P=S distance is only ∼0.934 that of P=Se (*i.e.*, 1.954/2.093 *vide supra*), had the relative sulfur occupancy been much lower, then a restraint to tie the P=Se/S distances *via* an FVAR parameter in *SHELXL* might have been necessary. A look at the list of ‘disagreeable’ reflections also shows an improvement (Tables 1 ▸–4 ▸ ▸ ▸). Note in particular that *none* of the worst offenders in Table 1 ▸ or 2 show up in Table 4 ▸. Based on these features and statistics, the structure for **5** shown in Fig. 6 ▸ is demonstrably the superior model for this crystal.

## Structural commentary

The structure of **5** (Fig. 6 ▸) shares many similarities with the di-sulfide **2** (structure **I** in Rawls *et al.*, 2023 ▸) and the di-selenide **3** (Jones *et al.*, 2015 ▸). As stated in section 2.4, in spite of the superpositional disorder of Se and S at both chalcogen sites, the unrestrained pnictogen-to-chalcogen bond distances [P1=Se1 = 2.0818 (8), P2=Se2 = 2.0879 (11), P1=S1 = 2.021 (19), P2=S2 = 1.953 (16) Å] are within or close to the normal ranges. All other bond distances and angles are also normal. There is a slight twist out of planarity at C1=C2, which gives a P1—C1—C2—P2 torsion angle of 9.0 (5)°. This, and torsion angles C9—P1—C1—C2 [−35.3 (3)°] and C1—C2—P2—C21 [−34. (3)°] effectively place phenyl rings C9–C14 and C12–C26 into an intra­molecular π–π-stacking arrangement. The dihedral angle between these overlapped phenyl rings is only 5.45 (3)° although the stacking is skewed, leading to a ring centroid–centroid distance of 3.737 (4) Å.

## Supra­molecular features

**Figure 7:**
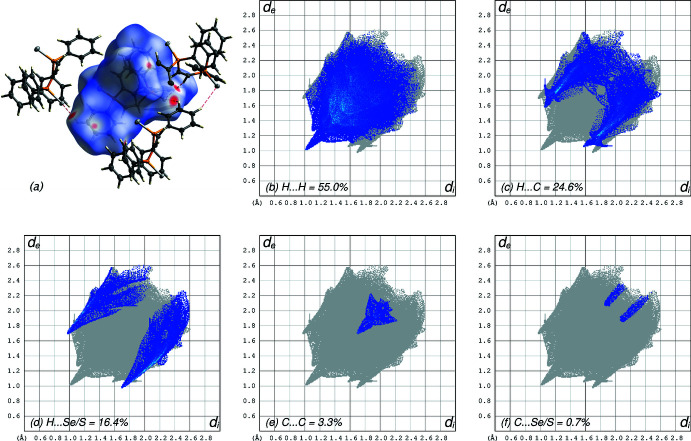
(*a*) A Hirshfeld-surface plot mapped over *d*_norm_ for the final model. Red spots and dashed lines highlight close contacts between C—H groups and the Se/S sites (see also Table 5 ▸). (*b*) Hirshfeld-surface fingerprint plot showing H⋯H contacts (55.0%). (*c*) Hirshfeld-surface fingerprint plot showing H⋯C contacts (24.6%). (*d*) Hirshfeld-surface fingerprint plot showing H⋯Se/S contacts (16.4%). (*e*) Hirshfeld-surface fingerprint plot showing C⋯C contacts (3.3%). (*f*) Hirshfeld-surface fingerprint plot showing C⋯Se/S contacts (0.7%).

The mol­ecular packing in **5** is similar to that in the di-sulfide **2** (Rawls *et al.*, 2023 ▸) and the di-selenide **3** (Jones *et al.*, 2015 ▸). Since all hydrogen atoms are bound to carbon, there are no strong hydrogen bonds. There are also no inter­molecular π–π inter­actions, though there are numerous weak C—H⋯π contacts. A Hirshfeld-surface analysis mapped over *d*_norm_ (Fig. 7 ▸) shows that inter­molecular contacts are dominated by hydrogen, either to other hydrogen atoms (55.0% of contacts), or to carbon (24.6%), or Se/S sites (16.4%). The remainder of the contacts (C⋯C at 3.3% and C⋯Se/S at 0.7%) are negligible. The strongest inter­actions, however, *i.e.* those in which distances are appreciably less than the sum of van der Waals radii (see intense red spots in Fig. 7 ▸ *a*) are from C—H⋯Se/S contacts (Table 5 ▸).

## Database survey

The Cambridge Structural Database (CSD, v5.43 with all updates through Nov. 2022; Groom *et al.*, 2016 ▸) returns 5727 entries for a search fragment consisting of the dppe mol­ecule. Of these, 895 have ‘any atom’ single bonded to the phospho­rus and 267 are double bonded. There are 35 entries with two P=S bonds and 17 with two P=Se bonds. There are also some mixed species; 33 entries have just one P=S and two entries have just one P=Se, though these mixed structures have little else in common with structure **5** discussed herein. The closest structures to **5** are the di-selenide structures YOWTIP (Jones *et al.*, 2015 ▸) and the di-sulfide CAMCUR01 (Rawls *et al.*, 2023 ▸).

## Refinement

A summary of data collection details and structure refinement statistics is given in Table 6 ▸. Hydrogen atoms were found in difference-Fourier maps, but subsequently included in the refinement using riding models, with constrained distances set to 0.95 Å. *U*_iso_(H) values were set to 1.2*U*_eq_ of the attached carbon atom. To ensure satisfactory refinement, constraints (*SHELXL* command EADP) were used to equalize displacement parameters of superimposed Se/S atoms.

## Conclusions

This crystal structure can serve as a straightforward instructional tool to demonstrate the varied pieces of information used to determine the quality and ultimately the correctness of a model. Here we investigated residual electron density, size of displacement ellipsoids, *F*_obs_^2^ and *F*_calc_^2^ mis-matches, plots of |*F*_obs_| *vs* |*F*_calc_| and comparison of inter­atomic distances to literature averages. Of particular importance is that the analysis highlights the dangers of uncritical suppression of outliers by inappropriate use of the OMIT command in *SHELXL*. Since the path to determining the best model inevitably varies from one structure to the next, a few additional points to consider, along with some background and, where appropriate, strategies to deal with them are included in the supporting information.

## Related literature

The supporting information includes a number of references that are not cited in the main paper. These sources are not exhaustive, but might serve as a useful starting point for further enquiry by an inter­ested student. They are grouped by their respective contexts and cited here:

*General advice on structure and refinement strategy*: Watkin, 1994 ▸; Clegg, 2019 ▸; Linden, 2020 ▸; Spek, 2020 ▸.

*Twinning*: Hahn & Klapper, 2006 ▸; Donnay & Donnay, 1959 ▸; Nespolo & Ferraris, 2003 ▸; Nespolo, 2015 ▸, 2019 ▸; Nespolo *et al.*, 2020 ▸; Herbst-Irmer & Sheldrick, 1998 ▸, 2002 ▸; Parsons, 2003 ▸; Parkin, 2021 ▸; Spek, 2020 ▸; Cooper *et al.*, 2002 ▸.

*Mol­ecular geometry and crystal symmetry*: Parkin, 1992 ▸; Allen *et al.*, 1987 ▸; Orpen *et al.*, 1989 ▸; Prince, 2006 ▸; Baur & Kassner, 1992 ▸; Marsh, 1997 ▸; Marsh & Spek, 2001 ▸; Le Page, 1987 ▸, 1988 ▸; Mohamed *et al.* (2016 ▸); Parkin *et al.*, 2023 ▸; Vinaya *et al.* (2023 ▸); Artioli *et al.* (1997 ▸); Parkin & Hope (1998 ▸).

*Rigid-body motion and TLS analysis*: Schomaker & Trueblood, 1968 ▸; Haestier *et al.*, 2008 ▸.

*Absorption correction*: de Meulenaer & Tompa, 1965 ▸; Blessing, 1995 ▸; Krause *et al.*, 2015 ▸.

*Extinction correction*: Darwin, 1914*a* ▸,*b* ▸; Becker & Coppens, 1974 ▸; Larson, 1967 ▸.

*SQUEEZE*: van der Sluis & Spek, 1990 ▸; Spek, 2015 ▸.

*Spherical scattering factor approximation*: Doyle & Turner, 1968 ▸; Dawson, 1964*a* ▸,*b* ▸; Coppens *et al.*, 1969 ▸.

*Multiple diffraction*: Renninger, 1937 ▸.

*λ/2 effects*: Kirschbaum *et al.*, 1997 ▸.

*Radiation damage*: Abrahams, 1973 ▸; Hope, 1975 ▸; Abrahams & Marsh, 1987 ▸; Moon *et al.*, 2011 ▸; Christensen *et al.*, 2019 ▸.

*Diffuse scatter and satellite reflections*: Bürgi, 2022 ▸; Stevens, 1974 ▸; Dornberger-Schiff, 1956 ▸; Zachariasen, 1967 ▸; Wagner & Schönleber, 2009 ▸; Petříček *et al.*, 2014 ▸.

## Supplementary Material

Crystal structure: contains datablock(s) I, global. DOI: 10.1107/S2056989023002700/dj2064sup1.cif

Structure factors: contains datablock(s) I. DOI: 10.1107/S2056989023002700/dj2064Isup2.hkl

Background, points to consider and strategies for determining the best model. DOI: 10.1107/S2056989023002700/dj2064sup3.pdf

the .res file for the mono-selenide model. DOI: 10.1107/S2056989023002700/dj2064sup4.txt

the .res file for the di-selenide model. DOI: 10.1107/S2056989023002700/dj2064sup5.txt

the .res file for the free occupancy selenide model. DOI: 10.1107/S2056989023002700/dj2064sup6.txt

the .res file for the mixed selenide/sulfide model. DOI: 10.1107/S2056989023002700/dj2064sup7.txt

CCDC reference: 2231833

Additional supporting information:  crystallographic information; 3D view; checkCIF report

## Figures and Tables

**Table 1:** List of the top four poorly fitting reflections for the model of the mono-selenide shown in Fig. 2 ▸

*h k l*	*F* ^2^_obs_	*F* ^2^_calc_	*error*/*s.u.*	*F*_calc_/*F*_calc(max)_	*d–spacing* (Å)
1 0 2	250.91	6.81	10.42	0.012	6.16
0 3 2	1787.72	504.88	10.20	0.101	3.74
2 0 3	335.44	41.35	9.52	0.029	3.76
1 4 0	671.93	175.18	8.75	0.060	3.18

All of the worst fitting reflections above have *F*_obs_^2^ >> *F*_calc_^2^ and *none* would be obscured by a well-designed beamstop.

**Table 2:** List of the top four poorly fitting reflections for the model of the di-selenide shown in Fig. 3 ▸

*h k l*	*F* ^2^_obs_	*F* ^2^_calc_	*error*/*s.u.*	*F*_calc_/*F*_calc(max)_	*d–spacing* (Å)
1 2 0	14663.16	2109.28	11.62	0.181	5.80
4 2 0	1787.72	13.29	10.10	0.014	2.78
0 4 3	335.44	114.61	10.00	0.042	2.71
0 2 0	671.93	1634.82	9.55	0.159	6.58

Three of the worst fitting reflections above have *F*_obs_^2^ >> *F*_calc_^2^ and *none* would be obscured by a well-designed beamstop.

**Table 3:** List of the top four poorly fitting reflections for the model of the mixed mono/di-selenide shown in Fig. 4 ▸

*h k l*	*F* ^2^_obs_	*F* ^2^_calc_	*error*/*s.u.*	*F*_calc_/*F*_calc(max)_	*d–spacing* (Å)
6 3 0	275.52	151.58	7.71	0.053	1.86
1 0 2	344.63	207.43	7.69	0.062	6.16
0 0 2	118.70	64.07	5.83	0.034	7.12
0 2 1	20.03	3.69	5.55	0.008	5.98

There are no egregious *F*_obs_^2^ >> *F*_calc_^2^ mis-matches for this model.

**Table 4:** List of the top four poorly fitting reflections for the model of the di-selenide shown in Fig. 5 ▸

*h k l*	*F* ^2^_obs_	*F* ^2^_calc_	*error*/*s.u.*	*F*_calc_/*F*_calc(max)_	*d–spacing* (Å)
6 3 0	270.87	165.41	7.11	0.055	1.86
0 8 0	301.14	215.46	5.07	0.063	1.65
0 6 2	784.43	965.40	4.86	0.133	2.10
2 5 1	57.90	29.40	4.80	0.023	2.39

There are no egregious *F*_obs_^2^ >> *F*_calc_^2^ mis-matches for this model.

**Table 5:** C—H⋯chalcogen close-contact geometry (Å, °)

*D*—H⋯*A*	*D*—H	H⋯*A*	*D*⋯*A*	*D*—H⋯*A*
C1—H1⋯Se2^i^	0.95	2.87	3.752 (13)	155.1
C1—H1⋯S2^i^	0.95	2.89	3.76 (2)	153.4
C8—H8⋯Se1	0.95	2.95	3.443 (8)	113.8
C8—H8⋯S1	0.95	2.82	3.325 (18)	114.5
C10—H10⋯Se1	0.95	2.92	3.459 (8)	117.4
C10—H10⋯S1	0.95	2.81	3.349 (19)	117.2
C20—H20⋯Se2	0.95	2.93	3.431 (13)	114.5
C20—H20⋯S2	0.95	2.92	3.40 (2)	112.6
C26—H26⋯Se2	0.95	3.00	3.524 (11)	116.6
C26—H26⋯S2	0.95	2.85	3.361 (17)	114.8

Symmetry code: (i) *x* + 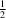 , −*y* + 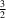 , −*z*.

**Table 6:** Experimental details

Crystal data	
Chemical formula	C_26_H_22_P_2_S_1.13_Se_0.87_
*M*_r_	501.46
Crystal system, space group	Orthorhombic, *P*2_1_2_1_2_1_
Temperature (K)	173
*a*, *b*, *c* (Å)	12.2833 (2), 13.1643 (2), 14.2478 (2)
*V* (Å^3^)	2303.88 (6)
*Z*	4
Radiation type	Cu *K*α
μ (mm^−1^)	4.32
Crystal size (mm)	0.49 × 0.45 × 0.34

Data collection	
Diffractometer	Bruker APEXII CCD
Absorption correction	Multi-scan (*SADABS*; Krause *et al.*, 2015 ▸)
*T*_min_, *T*_max_	0.587, 0.754
No. of measured, independent and observed [*I* > 2σ(*I*)] reflections	24246, 4187, 4155
*R*_int_	0.026
(sin θ/λ)_max_ (Å^−1^)	0.617

Refinement	
*R*[*F* ^2^ > 2σ(*F* ^2^)], *wR*(*F* ^2^), *S*	0.021, 0.053, 1.10
No. of reflections	4187
No. of parameters	279
H-atom treatment	H-atom parameters constrained
Δρ_max_, Δρ_min_ (e Å^−3^)	0.25, −0.23
Absolute structure	Flack *x* determined using 1619 quotients [(*I* ^+^)−(*I* ^−^)]/[(*I* ^+^)+(*I* ^−^)] (Parsons *et al.*, 2013 ▸).
Absolute structure parameter	0.018 (5)

Computer programs: *APEX2* (Bruker, 2013 ▸), *SHELXT* (Sheldrick, 2015*a* ▸), *SHELXL2019/2* (Sheldrick, 2015*b* ▸), *OLEX2* (Dolomanov *et al.*, 2009 ▸; Bourhis *et al.*, 2015 ▸), *Mercury* (Macrae *et al.*, 2020 ▸), *ShelXle* (Hübschle *et al.*, 2011 ▸), *CrystalExplorer* (Spackman *et al.*, 2021 ▸), *CrystalMaker* (Palmer, 2007 ▸), *SHELX* (Sheldrick, 2008 ▸) and *publCIF* (Westrip, 2010 ▸).

## supplementary crystallographic information

### Crystal data

**Table:**

C_26_H_22_P_2_S_1.13_Se_0.87_	*D*_x_ = 1.446 Mg m^−^^3^
*M**_r_* = 501.46	Cu *K*α radiation, λ = 1.54178 Å
Orthorhombic, *P*2_1_2_1_2_1_	Cell parameters from 9858 reflections
*a* = 12.2833 (2) Å	θ = 3.1–72.1°
*b* = 13.1643 (2) Å	µ = 4.32 mm^−^^1^
*c* = 14.2478 (2) Å	*T* = 173 K
*V* = 2303.88 (6) Å^3^	Block, yellow
*Z* = 4	0.49 × 0.45 × 0.34 mm
*F*(000) = 1023	

### Data collection

**Table:**

Bruker APEXII CCD diffractometer	4187 independent reflections
Radiation source: microsource	4155 reflections with *I* > 2σ(*I*)
Detector resolution: 7.41 pixels mm^-1^	*R*_int_ = 0.026
φ and ω scans	θ_max_ = 72.0°, θ_min_ = 4.6°
Absorption correction: multi-scan (*SADABS*; Krause *et al*., 2015)	*h* = −13→14
*T*_min_ = 0.587, *T*_max_ = 0.754	*k* = −15→16
24246 measured reflections	*l* = −17→17

### Refinement

**Table:**

Refinement on *F*^2^	Secondary atom site location: difference Fourier map
Least-squares matrix: full	Hydrogen site location: difference Fourier map
*R*[*F*^2^ > 2σ(*F*^2^)] = 0.021	H-atom parameters constrained
*wR*(*F*^2^) = 0.053	*w* = 1/[σ^2^(*F*_o_^2^) + (0.0259*P*)^2^ + 0.6437*P*] where *P* = (*F*_o_^2^ + 2*F*_c_^2^)/3
*S* = 1.10	(Δ/σ)_max_ < 0.001
4187 reflections	Δρ_max_ = 0.25 e Å^−^^3^
279 parameters	Δρ_min_ = −0.23 e Å^−^^3^
0 restraints	Absolute structure: Flack *x* determined using 1619 quotients [(*I*^+^)-(*I*^-^)]/[(*I*^+^)+(*I*^-^)] (Parsons *et al*., 2013).
Primary atom site location: structure-invariant direct methods	Absolute structure parameter: 0.018 (5)

### Special details

**Table:**

Geometry. All esds (except the esd in the dihedral angle between two l.s. planes) are estimated using the full covariance matrix. The cell esds are taken into account individually in the estimation of esds in distances, angles and torsion angles; correlations between esds in cell parameters are only used when they are defined by crystal symmetry. An approximate (isotropic) treatment of cell esds is used for estimating esds involving l.s. planes.
Refinement. Refinement progress was checked using *Platon* (Spek, 2020) and by an *R*-tensor (Parkin, 2000). The final model was further checked with the IUCr utility *checkCIF*.

### Fractional atomic coordinates and isotropic or equivalent isotropic displacement parameters (Å^2^)

**Table:**

	*x*	*y*	*z*	*U*_iso_*/*U*_eq_	Occ. (<1)
Se1	0.5609 (7)	0.3728 (6)	0.1269 (5)	0.0324 (6)	0.487 (3)
S1	0.5543 (16)	0.3748 (14)	0.1378 (11)	0.0324 (6)	0.513 (3)
Se2	0.3955 (11)	0.8180 (7)	−0.0416 (9)	0.0290 (8)	0.386 (3)
S2	0.3917 (16)	0.8053 (11)	−0.0401 (14)	0.0290 (8)	0.614 (3)
P1	0.53848 (5)	0.52619 (4)	0.15679 (4)	0.02193 (15)	
P2	0.44544 (5)	0.67007 (4)	−0.07209 (4)	0.02106 (15)	
C1	0.6036 (2)	0.60331 (18)	0.06839 (17)	0.0247 (5)	
H1	0.678166	0.615265	0.082046	0.030*	
C2	0.5726 (2)	0.64761 (18)	−0.01138 (17)	0.0238 (5)	
H2	0.632873	0.674300	−0.045092	0.029*	
C3	0.6118 (2)	0.56693 (18)	0.26141 (17)	0.0236 (5)	
C4	0.6416 (2)	0.6674 (2)	0.2747 (2)	0.0348 (6)	
H4	0.622485	0.717086	0.229226	0.042*	
C5	0.6993 (3)	0.6958 (2)	0.3544 (2)	0.0400 (7)	
H5	0.719472	0.764842	0.363164	0.048*	
C6	0.7273 (2)	0.6241 (2)	0.42085 (19)	0.0340 (6)	
H6	0.767453	0.643337	0.475021	0.041*	
C7	0.6967 (2)	0.5248 (2)	0.4079 (2)	0.0368 (7)	
H7	0.715302	0.475575	0.453854	0.044*	
C8	0.6394 (2)	0.4953 (2)	0.3293 (2)	0.0317 (6)	
H8	0.618731	0.426310	0.321502	0.038*	
C9	0.3993 (2)	0.56668 (18)	0.17259 (17)	0.0218 (5)	
C10	0.3177 (2)	0.4942 (2)	0.17614 (18)	0.0283 (6)	
H10	0.335274	0.424196	0.170070	0.034*	
C11	0.2103 (2)	0.5237 (2)	0.1886 (2)	0.0351 (6)	
H11	0.154201	0.474094	0.190052	0.042*	
C12	0.1851 (2)	0.6251 (2)	0.1987 (2)	0.0352 (6)	
H12	0.111475	0.645133	0.207103	0.042*	
C13	0.2660 (2)	0.6977 (2)	0.1968 (2)	0.0328 (6)	
H13	0.248108	0.767397	0.204846	0.039*	
C14	0.3735 (2)	0.6691 (2)	0.18307 (18)	0.0284 (6)	
H14	0.429257	0.719055	0.180830	0.034*	
C15	0.4883 (2)	0.65684 (19)	−0.19416 (17)	0.0249 (5)	
C16	0.5588 (2)	0.5791 (2)	−0.22096 (19)	0.0330 (6)	
H16	0.587585	0.533907	−0.175276	0.040*	
C17	0.5868 (2)	0.5682 (3)	−0.3148 (2)	0.0455 (8)	
H17	0.635568	0.515905	−0.333409	0.055*	
C18	0.5438 (3)	0.6331 (3)	−0.3812 (2)	0.0478 (8)	
H18	0.561813	0.624394	−0.445494	0.057*	
C19	0.4755 (3)	0.7097 (3)	−0.3548 (2)	0.0427 (8)	
H19	0.447116	0.754619	−0.400929	0.051*	
C20	0.4470 (2)	0.7225 (2)	−0.26115 (19)	0.0322 (6)	
H20	0.399383	0.775975	−0.243210	0.039*	
C21	0.3461 (2)	0.56962 (18)	−0.05739 (17)	0.0241 (5)	
C22	0.3769 (2)	0.46794 (19)	−0.06166 (18)	0.0308 (6)	
H22	0.451777	0.450049	−0.064155	0.037*	
C23	0.2976 (3)	0.3930 (2)	−0.0622 (2)	0.0379 (7)	
H23	0.318227	0.323468	−0.064779	0.045*	
C24	0.1892 (3)	0.4188 (2)	−0.0591 (2)	0.0431 (8)	
H24	0.135332	0.367069	−0.060500	0.052*	
C25	0.1579 (2)	0.5197 (3)	−0.0541 (2)	0.0434 (7)	
H25	0.082874	0.536992	−0.051575	0.052*	
C26	0.2364 (2)	0.5956 (2)	−0.05262 (19)	0.0308 (6)	
H26	0.215389	0.664886	−0.048398	0.037*	

### Atomic displacement parameters (Å^2^)

**Table:**

	*U*^11^	*U*^22^	*U*^33^	*U*^12^	*U*^13^	*U*^23^
Se1	0.0372 (10)	0.0261 (4)	0.0338 (16)	0.0063 (5)	0.0091 (10)	−0.0022 (9)
S1	0.0372 (10)	0.0261 (4)	0.0338 (16)	0.0063 (5)	0.0091 (10)	−0.0022 (9)
Se2	0.0335 (8)	0.017 (2)	0.0367 (4)	0.0043 (14)	−0.0011 (5)	−0.0014 (14)
S2	0.0335 (8)	0.017 (2)	0.0367 (4)	0.0043 (14)	−0.0011 (5)	−0.0014 (14)
P1	0.0201 (3)	0.0209 (3)	0.0248 (3)	0.0025 (2)	0.0022 (2)	0.0025 (2)
P2	0.0204 (3)	0.0208 (3)	0.0220 (3)	0.0002 (3)	0.0002 (2)	0.0000 (2)
C1	0.0179 (11)	0.0297 (13)	0.0266 (12)	−0.0028 (10)	0.0015 (10)	0.0006 (10)
C2	0.0188 (12)	0.0270 (12)	0.0257 (12)	−0.0018 (9)	0.0015 (9)	−0.0016 (9)
C3	0.0199 (12)	0.0272 (12)	0.0238 (12)	0.0052 (10)	0.002 (1)	0.0034 (10)
C4	0.0413 (16)	0.0288 (13)	0.0342 (14)	0.0031 (12)	−0.0110 (12)	0.0067 (11)
C5	0.0461 (17)	0.0342 (15)	0.0397 (16)	0.0021 (13)	−0.0112 (14)	0.0000 (13)
C6	0.0269 (13)	0.0500 (16)	0.0253 (13)	0.0059 (13)	−0.0039 (11)	0.0015 (12)
C7	0.0303 (14)	0.0482 (17)	0.0318 (14)	0.0078 (13)	−0.0021 (12)	0.0178 (13)
C8	0.0287 (14)	0.0316 (13)	0.0350 (14)	0.0038 (11)	0.0024 (12)	0.0119 (11)
C9	0.0195 (11)	0.0240 (11)	0.0219 (11)	0.0003 (10)	0.0022 (10)	0.0004 (9)
C10	0.0276 (13)	0.0263 (13)	0.0312 (13)	−0.0050 (11)	0.0045 (11)	−0.0004 (11)
C11	0.0237 (13)	0.0425 (16)	0.0393 (16)	−0.0091 (12)	0.0070 (12)	−0.0019 (13)
C12	0.0206 (13)	0.0506 (16)	0.0345 (14)	0.0065 (13)	0.0053 (11)	−0.0017 (13)
C13	0.0298 (15)	0.0299 (14)	0.0387 (15)	0.0083 (12)	0.0024 (12)	−0.0046 (11)
C14	0.0256 (13)	0.0267 (12)	0.0329 (13)	−0.0022 (11)	0.0002 (11)	−0.0014 (11)
C15	0.0196 (12)	0.0323 (14)	0.0227 (12)	−0.0076 (10)	−0.0006 (9)	−0.0024 (10)
C16	0.0255 (13)	0.0417 (14)	0.0318 (13)	−0.0013 (13)	−0.0001 (12)	−0.0085 (12)
C17	0.0290 (16)	0.064 (2)	0.0436 (18)	−0.0032 (15)	0.0073 (13)	−0.0202 (15)
C18	0.0373 (17)	0.080 (2)	0.0257 (14)	−0.0235 (18)	0.0070 (13)	−0.0069 (15)
C19	0.0452 (18)	0.0580 (19)	0.0250 (13)	−0.0177 (15)	−0.0022 (13)	0.0084 (13)
C20	0.0331 (14)	0.0324 (13)	0.0310 (13)	−0.0065 (12)	−0.0036 (13)	0.0025 (11)
C21	0.0245 (12)	0.0256 (12)	0.0223 (12)	−0.0031 (10)	0.0015 (10)	−0.001 (1)
C22	0.0333 (15)	0.0271 (13)	0.0321 (14)	0.0001 (11)	0.0062 (12)	−0.0013 (11)
C23	0.0496 (18)	0.0292 (14)	0.0348 (15)	−0.0089 (13)	0.0087 (13)	−0.0036 (12)
C24	0.0457 (18)	0.0461 (17)	0.0375 (16)	−0.0249 (15)	0.0081 (14)	−0.0064 (14)
C25	0.0254 (14)	0.0600 (19)	0.0448 (17)	−0.0114 (14)	0.0044 (13)	−0.0066 (15)
C26	0.0255 (13)	0.0344 (14)	0.0327 (14)	−0.0004 (11)	0.0045 (11)	−0.0032 (11)

### Geometric parameters (Å, º)

**Table:**

Se1—P1	2.082 (8)	C11—H11	0.9500
S1—P1	2.021 (19)	C12—C13	1.379 (4)
Se2—P2	2.088 (11)	C12—H12	0.9500
S2—P2	1.953 (16)	C13—C14	1.387 (4)
P1—C1	1.805 (2)	C13—H13	0.9500
P1—C9	1.805 (2)	C14—H14	0.9500
P1—C3	1.822 (3)	C15—C20	1.384 (4)
P2—C2	1.810 (2)	C15—C16	1.394 (4)
P2—C21	1.812 (2)	C16—C17	1.388 (4)
P2—C15	1.826 (2)	C16—H16	0.9500
C1—C2	1.333 (3)	C17—C18	1.379 (5)
C1—H1	0.9500	C17—H17	0.9500
C2—H2	0.9500	C18—C19	1.364 (5)
C3—C4	1.386 (4)	C18—H18	0.9500
C3—C8	1.392 (4)	C19—C20	1.390 (4)
C4—C5	1.389 (4)	C19—H19	0.9500
C4—H4	0.9500	C20—H20	0.9500
C5—C6	1.381 (4)	C21—C26	1.391 (4)
C5—H5	0.9500	C21—C22	1.392 (4)
C6—C7	1.373 (4)	C22—C23	1.387 (4)
C6—H6	0.9500	C22—H22	0.9500
C7—C8	1.378 (4)	C23—C24	1.375 (5)
C7—H7	0.9500	C23—H23	0.9500
C8—H8	0.9500	C24—C25	1.384 (5)
C9—C10	1.385 (4)	C24—H24	0.9500
C9—C14	1.393 (3)	C25—C26	1.389 (4)
C10—C11	1.386 (4)	C25—H25	0.9500
C10—H10	0.9500	C26—H26	0.9500
C11—C12	1.378 (4)		
			
C1—P1—C9	109.91 (11)	C12—C11—C10	119.9 (3)
C1—P1—C3	100.74 (12)	C12—C11—H11	120.0
C9—P1—C3	106.22 (11)	C10—C11—H11	120.0
C1—P1—S1	114.8 (5)	C11—C12—C13	120.5 (3)
C9—P1—S1	113.5 (6)	C11—C12—H12	119.7
C3—P1—S1	110.6 (6)	C13—C12—H12	119.7
C1—P1—Se1	110.1 (2)	C12—C13—C14	120.0 (3)
C9—P1—Se1	115.9 (2)	C12—C13—H13	120.0
C3—P1—Se1	112.8 (2)	C14—C13—H13	120.0
C2—P2—C21	114.01 (12)	C13—C14—C9	119.7 (2)
C2—P2—C15	100.99 (11)	C13—C14—H14	120.2
C21—P2—C15	103.59 (11)	C9—C14—H14	120.2
C2—P2—S2	109.2 (6)	C20—C15—C16	119.8 (2)
C21—P2—S2	114.3 (6)	C20—C15—P2	119.4 (2)
C15—P2—S2	114.0 (6)	C16—C15—P2	120.7 (2)
C2—P2—Se2	107.8 (4)	C17—C16—C15	119.6 (3)
C21—P2—Se2	117.3 (4)	C17—C16—H16	120.2
C15—P2—Se2	111.9 (4)	C15—C16—H16	120.2
C2—C1—P1	135.6 (2)	C18—C17—C16	120.1 (3)
C2—C1—H1	112.2	C18—C17—H17	120.0
P1—C1—H1	112.2	C16—C17—H17	120.0
C1—C2—P2	136.5 (2)	C19—C18—C17	120.3 (3)
C1—C2—H2	111.7	C19—C18—H18	119.8
P2—C2—H2	111.7	C17—C18—H18	119.8
C4—C3—C8	119.1 (2)	C18—C19—C20	120.6 (3)
C4—C3—P1	121.56 (19)	C18—C19—H19	119.7
C8—C3—P1	119.3 (2)	C20—C19—H19	119.7
C3—C4—C5	120.2 (3)	C15—C20—C19	119.6 (3)
C3—C4—H4	119.9	C15—C20—H20	120.2
C5—C4—H4	119.9	C19—C20—H20	120.2
C6—C5—C4	120.2 (3)	C26—C21—C22	120.1 (2)
C6—C5—H5	119.9	C26—C21—P2	118.59 (19)
C4—C5—H5	119.9	C22—C21—P2	120.9 (2)
C7—C6—C5	119.3 (3)	C23—C22—C21	119.6 (3)
C7—C6—H6	120.3	C23—C22—H22	120.2
C5—C6—H6	120.3	C21—C22—H22	120.2
C6—C7—C8	121.2 (3)	C24—C23—C22	120.2 (3)
C6—C7—H7	119.4	C24—C23—H23	119.9
C8—C7—H7	119.4	C22—C23—H23	119.9
C7—C8—C3	119.8 (3)	C23—C24—C25	120.5 (3)
C7—C8—H8	120.1	C23—C24—H24	119.7
C3—C8—H8	120.1	C25—C24—H24	119.7
C10—C9—C14	119.9 (2)	C24—C25—C26	119.9 (3)
C10—C9—P1	119.12 (19)	C24—C25—H25	120.1
C14—C9—P1	120.98 (19)	C26—C25—H25	120.1
C9—C10—C11	120.0 (2)	C25—C26—C21	119.7 (3)
C9—C10—H10	120.0	C25—C26—H26	120.2
C11—C10—H10	120.0	C21—C26—H26	120.2
			
C9—P1—C1—C2	−35.3 (3)	C11—C12—C13—C14	1.0 (4)
C3—P1—C1—C2	−147.1 (3)	C12—C13—C14—C9	−0.9 (4)
S1—P1—C1—C2	94.1 (7)	C10—C9—C14—C13	−0.2 (4)
Se1—P1—C1—C2	93.6 (4)	P1—C9—C14—C13	−178.8 (2)
P1—C1—C2—P2	9.0 (5)	C2—P2—C15—C20	−140.0 (2)
C21—P2—C2—C1	−34.1 (3)	C21—P2—C15—C20	101.8 (2)
C15—P2—C2—C1	−144.5 (3)	S2—P2—C15—C20	−23.0 (7)
S2—P2—C2—C1	95.1 (6)	Se2—P2—C15—C20	−25.5 (4)
Se2—P2—C2—C1	98.1 (5)	C2—P2—C15—C16	42.5 (2)
C1—P1—C3—C4	38.4 (3)	C21—P2—C15—C16	−75.8 (2)
C9—P1—C3—C4	−76.2 (2)	S2—P2—C15—C16	159.5 (6)
S1—P1—C3—C4	160.2 (6)	Se2—P2—C15—C16	156.9 (4)
Se1—P1—C3—C4	155.8 (3)	C20—C15—C16—C17	−0.1 (4)
C1—P1—C3—C8	−141.1 (2)	P2—C15—C16—C17	177.4 (2)
C9—P1—C3—C8	104.3 (2)	C15—C16—C17—C18	−0.9 (4)
S1—P1—C3—C8	−19.3 (6)	C16—C17—C18—C19	1.4 (5)
Se1—P1—C3—C8	−23.7 (3)	C17—C18—C19—C20	−0.9 (5)
C8—C3—C4—C5	0.7 (4)	C16—C15—C20—C19	0.6 (4)
P1—C3—C4—C5	−178.8 (2)	P2—C15—C20—C19	−177.0 (2)
C3—C4—C5—C6	0.0 (5)	C18—C19—C20—C15	0.0 (4)
C4—C5—C6—C7	−0.7 (5)	C2—P2—C21—C26	145.1 (2)
C5—C6—C7—C8	0.6 (4)	C15—P2—C21—C26	−106.1 (2)
C6—C7—C8—C3	0.1 (4)	S2—P2—C21—C26	18.5 (7)
C4—C3—C8—C7	−0.8 (4)	Se2—P2—C21—C26	17.7 (5)
P1—C3—C8—C7	178.8 (2)	C2—P2—C21—C22	−42.3 (2)
C1—P1—C9—C10	133.8 (2)	C15—P2—C21—C22	66.5 (2)
C3—P1—C9—C10	−118.1 (2)	S2—P2—C21—C22	−168.9 (7)
S1—P1—C9—C10	3.7 (6)	Se2—P2—C21—C22	−169.7 (4)
Se1—P1—C9—C10	8.1 (3)	C26—C21—C22—C23	0.8 (4)
C1—P1—C9—C14	−47.5 (2)	P2—C21—C22—C23	−171.8 (2)
C3—P1—C9—C14	60.6 (2)	C21—C22—C23—C24	0.4 (4)
S1—P1—C9—C14	−177.6 (5)	C22—C23—C24—C25	−0.9 (5)
Se1—P1—C9—C14	−173.2 (3)	C23—C24—C25—C26	0.4 (5)
C14—C9—C10—C11	1.1 (4)	C24—C25—C26—C21	0.7 (4)
P1—C9—C10—C11	179.8 (2)	C22—C21—C26—C25	−1.3 (4)
C9—C10—C11—C12	−0.9 (4)	P2—C21—C26—C25	171.4 (2)
C10—C11—C12—C13	−0.1 (4)		

### Hydrogen-bond geometry (Å, º)

**Table:**

*D*—H···*A*	*D*—H	H···*A*	*D*···*A*	*D*—H···*A*
C1—H1···Se2^i^	0.95	2.87	3.752 (13)	155
C1—H1···S2^i^	0.95	2.89	3.76 (2)	153
C8—H8···Se1	0.95	2.95	3.443 (8)	114
C8—H8···S1	0.95	2.82	3.325 (18)	115
C10—H10···Se1	0.95	2.92	3.459 (8)	117
C10—H10···S1	0.95	2.81	3.349 (19)	117
C20—H20···Se2	0.95	2.93	3.431 (13)	115
C20—H20···S2	0.95	2.92	3.40 (2)	113
C26—H26···Se2	0.95	3.00	3.524 (11)	117
C26—H26···S2	0.95	2.85	3.361 (17)	115

Symmetry code: (i) *x*+1/2, −*y*+3/2, −*z*.
