# Supplementary material for: A mixed phosphine sulfide/selenide structure as an instructional example for how to evaluate the quality of a model
Source: Acta Crystallogr E Crystallogr Commun. 2023 Mar 28;79(Pt 4):246–53. doi: 10.1107/S2056989023002700 (PMC10088320; doi:10.1107/S2056989023002700)
Supplement: Supplementary file 3 [file e-79-00246-sup3.pdf]

Supplementary Information for

A mixed phosphine sulfide/selenide structure as an instructional example for how to evaluate the quality of a model

Structure problems may show up in the diffraction data (*i.e.*, in reciprocal space) or in the structure model (real space). This supplementary information serves to highlight a few problems that may arise and, where appropriate, to suggest explanations and/or remedies. Similar analyses have been given before [see for example, Watkin (1994), Clegg (2019), Linden (2020), Spek, 2020] in varying levels of detail. The intent is to provide the student with a ready list of phenomena to be aware of and the instructor with a series of talking points. To that end, references are included that a student might turn to for further information.

S1. Reflections with $F_{obs}^2 << F_{calc}^2$

In most cases, this type of disagreement has a straightforward cause that can be identified. If reflections with large discrepancies of this type meet specific criteria (*viz.*, error/SU > 10), an alert in the *checkCIF* report may appear (PLAT918). Four scenarios are described below, along with strategies to diagnose the source.

S1.1. *Reflections obscured by the beamstop*
A common reason for an observed reflection (F_{obs}^2) to be less intense than expected is that it is obscured or partially truncated by the beamstop. For this to be the case, the reflections must be at low diffraction angle because the shadow cast by a typical beamstop only extends out to ~8 to 10 Å or so. For instance, a reflection with indices (0 0 1) at a resolution (approximated as the Bragg *d*-spacing) of (say) ~11 Å would be a candidate. In this case, use of the OMIT command would be warranted because the poor fit has a known cause that cannot be easily rectified. Indeed, for genuine cases of reflections obscured by the beamstop, retaining the affected reflection would be worse than omitting it. In the vast majority of cases this represents the most justifiable cause for using OMIT on individual reflections.

S1.2. *Reflections obscured by the beamstop support*
Vertical beamstop supports (plus horizontal supports in some dual-wavelength diffractometers) can also block, or partially block individual reflections. However, a typical data collection strategy involves multiple recordings of each reflection (or symmetry equivalents) over many different regions of the detector. Thus, for any such obscured reflection there are invariably a few equivalents that are unaffected. All modern area-detector integration and scaling/merging routines [*e.g*. *CrysAlis PRO* (Rigaku-OD, 2017); *Apex2/3/4* (Bruker-AXS, 2016; *X-AREA* (Stoe & Cie, 2002) *etc*.] should automatically filter out such bad measurements. For suspect cases, facilities exist to locate individual measurements on particular frames. If necessary, any individual bad measurement(s) may be removed manually, leaving the rest [either identical (h k l) or symmetry equivalent(s)] intact.

S1.3. *Reflections clipped at the detector edge*
Some reflections will inevitably be clipped by the edge of the detector, and therefore return fewer counts than expected. As with the case in A1.2 above, a suitable data collection strategy would (by default) anticipate this situation, especially for data collections employing multiple detector 2θ angles, as is generally the case for longer wavelengths (typically Cu$K\alpha$). As such, the data reduction software should filter out affected measurements. If in doubt, suspect measurements should be inspected, as per S1.2, above.

S1.4. *Intense reflections that overload the detector*
For strongly diffracting crystals, some reflections may be too intense and thereby overload the detector. How this manifests in practice is detector and/or software specific. Affected reflections may be recorded as less intense than expected due to maxing out the detector. One common solution is to record a 'fast' scan, from which overloads in the main scans may be substituted. Other solutions include attenuator insertion, lower X-ray generator power, or a smaller crystal. Potential cases should preferably be identified during data collection setup.

S2. Reflections with F_{obs}^2 >> F_{calc}^2

When $F_{obs}^2 >> F_{calc}^2$, the experimental data include information that is not accounted for by the model. Such differences between F_{obs}^2 and F_{calc}^2 include information on any model deficiencies. There is little, if any, scientific justification to use OMIT on affected reflections. Such problems have a number of potential causes, which should be identified and dealt with. Uncritical cherry-picking of ill-fitting datapoints (outliers) should be strictly avoided.

S2.1. *Twinning*

There is an extensive literature on twinning (see *e.g*. Hahn & Klapper, 2006 and references therein). Without going into unnecessary detail, a twinned crystal consists of two or more components related by a well-defined mathematical operation (Donnay & Donnay, 1959; Nespolo & Ferraris, 2003; Nespolo, 2015, 2019; Herbst-Irmer & Sheldrick, 1998, 2002; Parsons, 2003). A concise summary was recently given by Parkin (2021). For molecular crystals, this is typically a non-crystallographic two-fold rotation. For the current purpose, there are two main distinctions: cases where reciprocal lattices only partially overlap (as in non-merohedric twins), and cases of complete (or near complete) overlap [as in twinning by (pseudo-)merohedry]. The former is usually readily apparent from initial diffraction images and facilities exist within all modern diffractometer software to index separate components and treat accordingly. For twinning by merohedry (more common for inorganics) or pseudo-merohedry (common for organics and organometallics), complete (or near complete) reciprocal lattice overlap often hides twin identification until after structure solution/refinement problems arise. Nevertheless, in many cases twin operations may be apparent from the unit cell metrics [*e.g.*, monoclinic with beta ~90° or ~120° (Nespolo *et al.*, 2020), but see Herbst-Irmer & Sheldrick (1998) for a more comprehensive list]. For a twinned crystal, if twinning is not included in the model, then the F_{calc}^2 values will contain substantial contribution from second (or higher) components. Such differences are exploited by programs such as *TwinRotMat* in *PLATON* (Spek, 2020) and *Rotax* (Cooper *et al.*, 2002) to derive potential twin operations. Once the correct twin law is identified and an appropriate twin operation included in the refinement model, the correspondence between F_{obs}^2 and F_{calc}^2 is often as good as that of non-twinned crystals.

S2.2. *Aggregate or composite crystals*

Aggregates are composed of two or more pieces that are *not* related by a valid twin operation. Examples include crystals that crashed out of solution resulting in a shower of randomly intergrown pieces, cracked crystals, *etc*. The best solution is to find, break, or to cut away a single-crystal fragment. If this is not possible, in many cases the largest parts of an aggregate may be indexed separately and treated using the same software tools designed for non-merohedric twins. Depending on the severity, some cracked crystals might yield datasets with low-angle reflections having contributions from all fragments, while higher angle reflections might be predominantly from just the largest. In such cases, removal of a small number of the worst offenders might be justified, provided a sufficiently detailed explanation is also given, *e.g.*, in a *checkCIF* VRF (Validation Reply Form).

S2.3. *Structure composition errors*

As exemplified by the mono-Se and di-Se preliminary trial models for structure **5** given in the accompanying paper, simple mis-identification of element types can give large $F_{calc}^2 >> F_{obs}^2$ mismatches. Once the actual cause of the poor correspondence between F_{calc}^2 and F_{obs}^2 is rectified, the fit invariably becomes more than satisfactory. A well-known case of geometry problems caused by incorrect elemental composition was seen in the bond-stretch isomer fiasco mentioned in section 2.4, above (Parkin, 1992).

S3. Other mismatches of F_{obs}^2 and F_{calc}^2

S3.1. *Mis-assigned symmetry*

The modern structure analyst has at their disposal, many decades of accumulated knowledge of molecular and crystal structure. In short, it is exceedingly unlikely for bond lengths and angles to deviate substantially from their typical literature values (*e.g.*, Allen *et al*., 1987; Orpen *et al*., 1989; Prince, 2006). Perhaps the single most common cause of geometry problems, at least historically, has been incorrect space group assignment, for which many cases have been documented (*e.g.*, Baur & Kassner, 1992; Marsh, 1997; Marsh & Spek, 2001). Nowadays, due to symmetry checking (*e.g.*, Le Page, 1987; 1988) in the validation routines of *PLATON* (Spek, 2020) and the ubiquity

of IUCr *checkCIF*, such problems are much less common. Geometry problems also occur for cases of incomplete disorder modelling [*e.g*.. Mohamed *et al*. (2016) *cf* Parkin *et al*. (2023)], or missed twinning [*e.g.*, Artioli *et al*. (1997) *vs* Parkin & Hope (1998)].

S3.2. *Inadequate absorption correction*
Easily applied correction algorithms [*e.g*., *via* face indexing (de Meulenaer & Tompa, 1965) and by multi-scan methods (Blessing, 1995)] are now fully integrated into all diffractometer manufacturer software. Except in the most severe cases, absorption is essentially a solved problem. Nevertheless, such corrections must be applied with care. For example, to account for the resolution-dependent component of absorption, multi-scan methods require an estimate of the average crystal radius (if it were assumed to be a sphere). For the implementation in *SADABS* (Krause *et al.*, 2015), the recommendation is to bias this estimate towards the smallest crystal dimension. Taken at face value, the example given (in Krause *et al*., 2015) up-weights the smallest dimension by ~5.5 times, an approximation that works *very* well over a wide range of crystal sizes and compositions.

S3.3. *Extinction*
The symptoms of extinction are readily identified as systematically smaller F_{obs}^2 compared to F_{calc}^2 for the strongest reflections in a *SHELXL* *.lst table of "most disagreeables". Extinction comprises two separate phenomena [see Darwin (1914a,b)] that attenuate strong reflections. Although their mechanisms are distinct (see *e.g.*, Becker & Coppens, 1974), the effect on diffracted intensities is similar. Thus, in practice a single empirical correction is applied, generally by some variant of the approximation formulated by Larson (1967). In general, if the correction (*e.g*., EXTI in *SHELXL*) is greater than about 3 times its standard uncertainty, then the correction is retained.

S4. Additional problems to be aware of

S4.1*. The spherical scattering factor approximation*
The vast majority of routine structure determinations make use of spherically symmetric atomic scattering factors (see *e.g*., Doyle & Turner, 1967). For light atoms in molecules, however, the valence electron distribution is decidedly non-spherical. The approximation leads to limits on atomic coordinate *accuracy* of as much as 0.02 Å, no matter what the *precision* might be (Dawson, 1964a,b; Coppens *et al*., 1969).

S4.2*. Rigid-body motions*
Rigid body motion in crystal structures comprises translation (T), libration (or rotary oscillation, L), and screw (S) motions, which may be modelled by a combination of tensors (TLS analysis, Schomaker & Trueblood, 1968). The effects can lead to errors in bond lengths of as much as a few hundredths of an ångström (Haestier *et al.*, 2008). The effects are diminished for low-temperature data.

S4.3*. Multiple diffraction and Renninger effects*
Multiple diffraction may occur if two reciprocal lattice points are simultaneously in a position to diffract (*i.e*., on the Ewald sphere surface). In effect, the diffracted beam for one reflection acts as a primary beam for the second, augmenting its intensity. Such double reflections (Renninger, 1937) tend to be weak but can, for example, populate systematically absent reflections with appreciable intensity, thereby confusing glide plane and/or screw axis assignment.

S4.4. *Thermal diffuse scatter*
Bulk lattice vibrations, *i.e*., optical and acoustic phonons *etc.*, may affect intensities *via* thermal diffuse scattering, which affects the overall scattered background, but also peaks at the positions of diffraction maxima (see *e.g.* Bürgi, 2022). In some instances, corrections have been formulated [*e.g*., Stevens (1974) and references therein]. Such problems may sometimes be minimized by collecting data at low temperature.

S4.5. *Other diffraction features*
Crystal structure defects that serve to lower the periodicity, such as order-disorder phenomena (*e.g.* Dornberger-Schiff, 1956), stacking faults (*e.g.*, Zachariasen, 1967), etc. may lead to streaks or other diffuse scatter between Bragg maxima. Conversely, incommensurately modulated structures [see Wagner & Schönleber (2009) for a gentle

introduction] exhibit additional satellite reflections clustered about Bragg maxima. Full treatment of such effects usually require appropriate refinement software *e.g. JANA* (Petříček *et al*., 2014)

S4.6. *λ/2 effects*
Monochromators are not perfect. For a given characteristic X-ray line (*e.g.* Mo$K\alpha_{1,2}$) at normal operating voltage there will be some small amount of the bremsstrahlung radiation with a wavelength exactly half that of the characteristic $K\alpha_{1,2}$ emissions. The reason is clear from Bragg's law: diffraction from (hkl) for X-rays of wavelength λ occurs at the same diffraction angle 2θ as diffraction for $\lambda/2$ X-rays for reflection (2h,2k,2l). The effects have been investigated by Kirschbaum *et al.* (1997), who concluded that the effects are too small to affect routine structures but might be important for charge-density studies.

S4.7. *Radiation damage*
Radiation damage is a common source of intensity errors, particularly for room temperature data (Abrahams, 1973), the effects being position and time dependent. Intensity changes are typically negative, though for very intense reflections changes, can be positive (Hope, 1975). Methods of correction have been available for many years (*e.g.*, Abrahams & Marsh, 1987). For small molecules, low-temperature data collection is usually, though not always, an effective solution (see *e.g*., Moon *et al*., 2011; Christensen *et al*., 2019)

S4.8. *Consequences of SQUEEZE*
The *SQUEEZE* routine (van der Sluis & Spek, 1990; Spek, 2015) is invaluable for factoring out the effects of intractable solvent disorder. Its effect on differences between F_{obs}^2 and F_{calc}^2, however, can be unpredictable, in some cases giving large apparent mismatches between observed and calculated data. In such cases, our own preference is to retain ill-fitting datapoints and let the reader decide. Any resulting *checkCIF* alerts may be explained in a VRF such as “There is no obvious reason why these reflections might have been recorded improperly, but perhaps the mis-match is related to the use of *SQUEEZE*. We prefer not to omit data without a valid scientific reason, which is why these data points remain in the dataset.”

References

Abrahams, S. C. (1973). *Acta Cryst*. A**29**, 111–116.

Abrahams, S. C. & Marsh, P. (1987). *Acta Cryst*. A**43**, 265–269.

Allen, F.H., Kennard, O., Watson, D.G., Brammer, L., Orpen, A.G. & Taylor, R. (1987). *J. Chem. Soc., Perkin Trans*., S1–S19.

Artioli, G., Masciocchi, N. & Galli, E. (1997). *Acta Cryst*. B**53**, 498–503.

Baur, W. H. & Kassner, D. (1992). *Acta Cryst*. B**48**, 356–369.

Becker, P. J. & Coppens, P. (1974). *Acta Cryst*. A**30**, 129–147.

Bürgi, H.-B. (2022). *Acta Cryst*. B**78**, 283–289.

Blessing, R.H. (1995). *Acta Cryst*. A**51**, 33–38.

Christensen, J., Horton, P. N., Bury, C. S., Dickerson, J. L., Taberman, H., Garman, E. F. & Coles, S. J. (2019). *IUCrJ* **6**, 703--713.

Coppens, P., Sabine, T.M., Delaplane, R.G. & Ibers, J.A. (1969). *Acta Cryst*. B**25**, 2451–2458.

Cooper, R. I., Gould, R. O., Parsons, S. & Watkin, D. J. (2002). *J. Appl. Cryst*. **35**, 168--174.

Darwin, C.G. (1914a). *Phil. Mag*. **27**, 315 –333.

Darwin, C.G. (1914b). *Phil. Mag*. **27**, 675 –690.

Dawson, B. (1964a). *Acta Cryst*. **17**, 990–996.

Dawson, B. (1964b). *Acta Cryst*. **17**, 997–1009.

Donnay, J. D. H & Donnay, G. (1959). *International Tables for X-ray Crystallography, vol. II*, p104. Birmingham, Kynoch Press.

Dornberger-Schiff, K. (1956). *Acta Cryst*. **9**, 593–601.

Doyle, P.A. & Turner, P.S. (1968). *Acta Cryst.* A2**4**, 390–397.

Haestier, J., Sadki, M., Thompson, A. L. & Watkin, D. (2008). *J. Appl. Cryst*. **41**, 531–536.

Hahn, Th. & Klapper, H. (2006). *International Tables for Crystallography, vol. D*, 393–448.

Herbst-Irmer, R. & Sheldrick, G. M. (1998). *Acta Cryst*. B**54**, 443–449.

Herbst-Irmer, R. & Sheldrick, G. M. (2002). *Acta Cryst*. B**58**, 477–481.

Hope, H. (1975) in "*Anomalous Scattering*" Ramaseshan & Abrahams, *eds*. IUCr, Munksgaard, Copenhagen

Kirschbaum, K., Martin, A. & Pinkerton, A. A. (1997). *J. Appl. Cryst*. **30**, 514–516.

Larson, A. C. (1967). *Acta Cryst*. **23**, 664–665.

de Meulenaer, J. & Tompa, H. (1965). *Acta Cryst*. **19**, 1014–1018.

Marsh, R. E. & Spek, A. L. (2001). *Acta Cryst*. B**57**, 800–805.

Moon, D.K, Tripathi, A., Sullivan, D., Siegler, M.A., Parkin, S. & Posner, G.H. (2011). *Bioorg. Med. Chem. Lett*., **21**, 2773–2775.

Nespolo, M. (2015). *Cryst. Res. Technol*. **50**, 362–371.

Nespolo, M. (2019). *Acta Cryst*. A**75**, 551–573.

Nespolo, M. & Ferraris, G. (2003). Z. Kristallogr. **218**, 178–181.

Nespolo, M., Smaha, R. W. & Parkin, S. (2020). *Acta Cryst*. B**76**, 643–649.

Orpen, A.G., Brammer, L., Allen, F.H., Kennard, O., Watson, D.G. & Taylor, R. (1989). J. Chem. Soc., Dalton Trans., S1–S83.

Parkin, S. & Hope, H. (1998). *Acta Cryst*. B**54**, 339–344.

Parsons, S. (2003). *Acta Cryst*. D**59**, 1995–2003.

Petříček, V., Dušek, M. & Plášil, J. (2014). *Z. Kristallogr.* **229**, 345–352.

Renninger, M. (1937). *Z. Phys.* **106**, 141–176.

Schomaker, V. & Trueblood, K. N. (1968). *Acta Cryst.* B**24**, 63–76.

Spek, A. L. (2015). *Acta Cryst.* C**71**, 9–18.

Sluis, P. van der & Spek, A. L. (1990). *Acta Cryst.* A**46**, 194–201.

Wagner, T. & Schönleber, A. (2009). *Acta Cryst.* B**65**, 249–268.

Zachariasen, W. H. (1967). *Acta Cryst.* **23**, 44–49.
